# Hemoptysis in a 48-Year-Old Man

**DOI:** 10.1016/j.chpulm.2026.100275

**Published:** 2026-06-11

**Authors:** Shannon Tseng, Angela Montes, Stephanie Rothweiler, Bonnie Garon, Alison Wilcox, Wafaa Elatre, Rodrigo Garcia Tome, Patrick Chan

**Affiliations:** a Department of Internal Medicine, Los Angeles General Medical Center, University of Southern California, Los Angeles, CA; b Division of Pulmonary and Critical Care Medicine, Department of Medicine, Los Angeles General Medical Center, University of Southern California, Los Angeles, CA; c Cardiothoracic Imaging Section, Department of Radiology, Los Angeles General Medical Center, Los Angeles, CA; d Department of Pathology, University of Southern California, Los Angeles, CA

## Case Presentation

**Figure 1:**
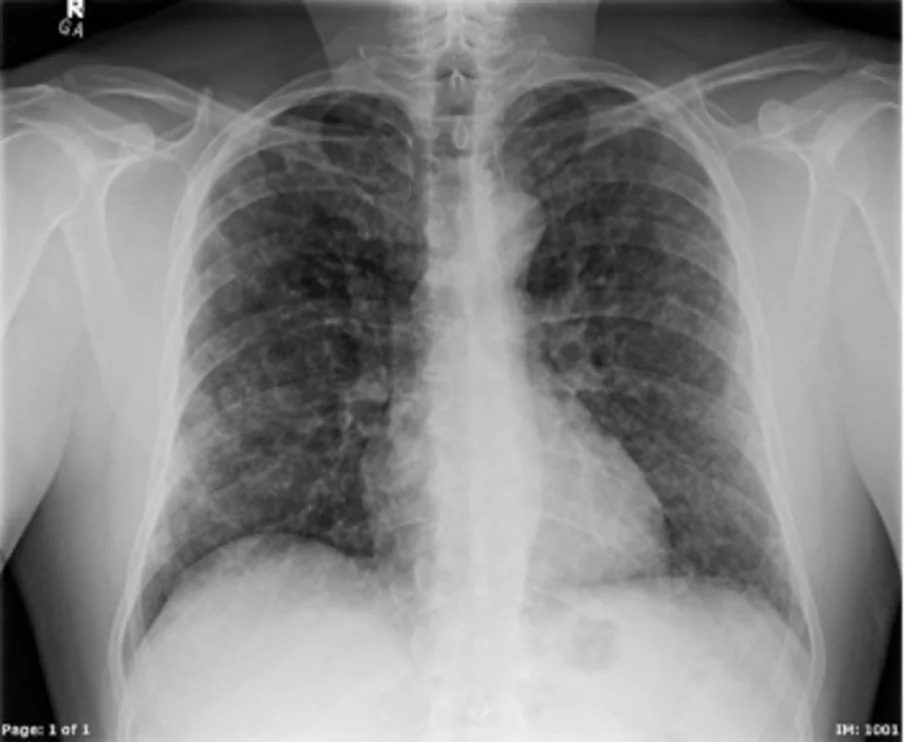
Upright posteroanterior chest radiograph demonstrated diffuse nodular opacities.

A 48-year-old man with no medical history presented with a 2-week history of scant hemoptysis. He had no fever, cough, shortness of breath, weight loss, night sweats, sick contacts, recent travel, or known TB exposure. He had no history of prior hospitalization, incarceration, or recurrent respiratory infections. He was born in Mexico but moved to Los Angeles, California, around 21 years of age. His primary care doctor obtained a chest radiograph that revealed abnormal findings (Fig 1). He was advised to present to the emergency department for further evaluation.

**Figure 2:**
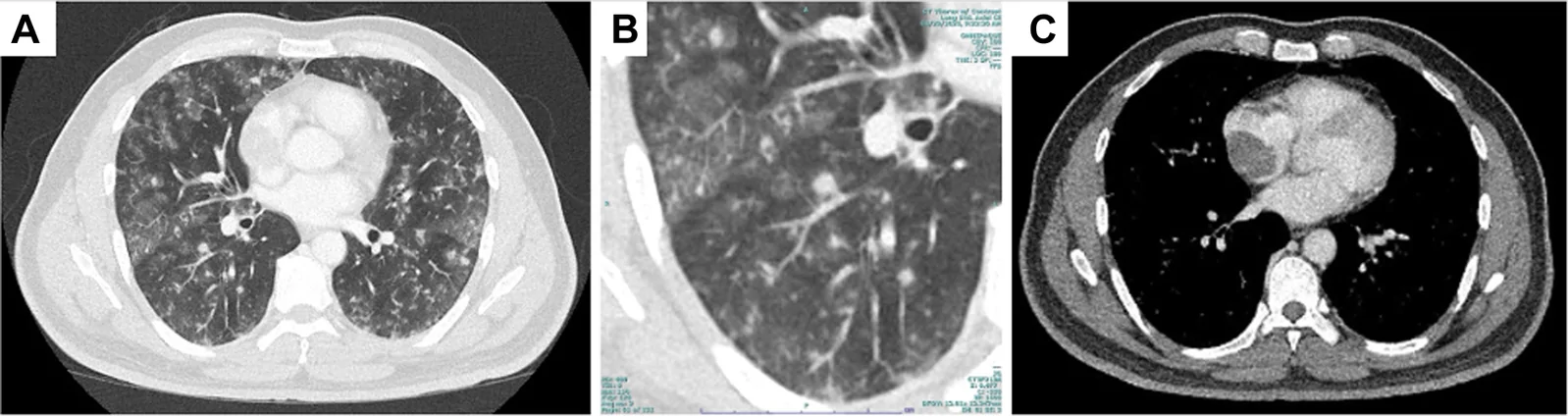
A, Chest CT scan in 3-mm slices revealed patchy ground-glass opacities with superimposed solid micronodules. Coned-down view of the right lower lobe showed solid nodules arranged in a tree-in-bud distribution. B, Most ground-glass opacities were centrilobular. C, Incidental finding of a right-sided atrial mass was seen in the soft tissue window.

On initial examination, his temperature was 36.9 °C with a heart rate of 97 beats/min and BP of 137/89 mm Hg. His peripheral oxygen saturation was 97% on room air. Cardiac examination was unremarkable. He had normal work of breathing with diffuse crackles in the bilateral lung fields. Laboratory studies revealed a leukocyte count of 6,000 cells/μL, hemoglobin level of 12.5 g/dL, and platelet count of 204,000 cells/μL. Broad infectious and autoimmune workup was initiated. CT scan of the chest was obtained (Fig 2).

**Figure 3:**
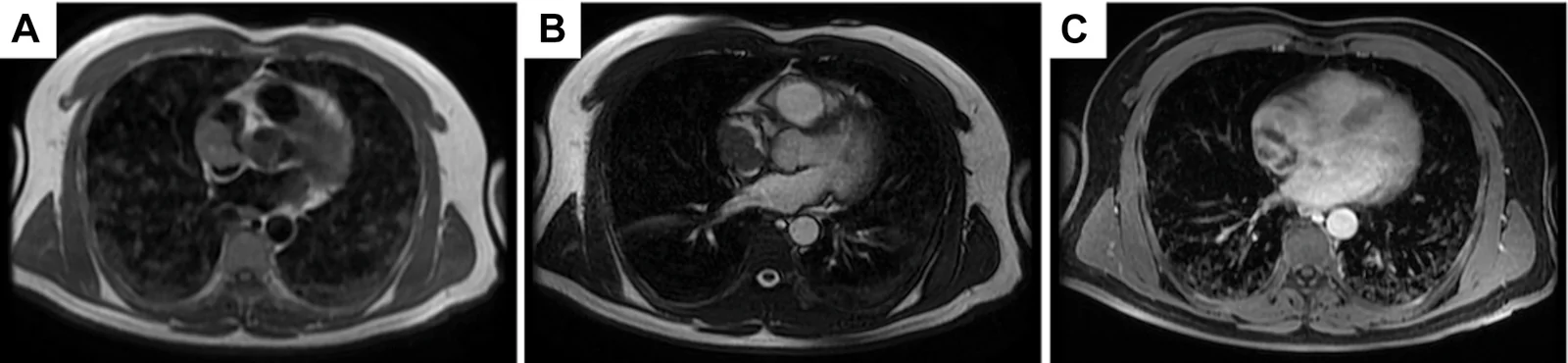
A-C, Cardiac MRI scan in 8-mm slices with black blood images (A) and fast imaging using steady-state acquisition short-axis images (B) demonstrated minimal heterogeneity in signal centrally. C, Postgadolinium enhancement was also heterogeneous centrally, consistent with a solid enhancing mass with likely some associated peripheral hemorrhage.

Transthoracic echocardiogram and cardiac MRI scan were obtained to better characterize the right-sided atrial mass. Transthoracic echocardiogram showed a nonmobile, heterogenous, and irregular mass that appeared adherent to the right atrial posterior wall with a small mobile component. Cardiac MRI scan characterized the mass as heterogenous and centrally enhancing with lobulated borders (Fig 3).

A multidisciplinary discussion was held with pulmonology, cardiology, internal medicine, and radiology to determine the optimal method of biopsy. Ultimately, bronchoscopy with transbronchial biopsy was preferred over cardiac biopsy because of the risk of embolization and risk of obtaining a nondiagnostic cardiac biopsy given the presence of surrounding thrombus. The patient underwent bronchoscopy with bronchoalveolar lavage, transbronchial cryobiopsy, and endobronchial ultrasound. Cryobiopsy revealed tumor cells with angiocentric growth and neoplastic cells lining vascular spaces. Immunohistochemistry was positive for vascular markers, including cluster of differentiation 31 and erythroblast transformation-specific related gene, and was negative for cytokeratin and thyroid transcription factor-1.

*What is the diagnosis?*

*Diagnosis:* Epithelioid hemangioendothelioma presenting as a cardiac mass with pulmonary metastasis

## Discussion

### Clinical Discussion

The patient’s chief complaint was hemoptysis. The differential for hemoptysis often includes infection, autoimmune process, and malignancy. TB is the leading cause of hemoptysis worldwide. In the US, the most common cause of hemoptysis is acute bronchitis.^1^ Given the acute onset of his hemoptysis and origin from an endemic region, infectious etiology was high on the differential. This etiology was also consistent with CT imaging findings of tree-in-bud (TIB) nodularity and centrilobular nodules. However, given the lack of systemic symptoms, productive cough, and leukocytosis, the presentation was atypical for an infectious etiology despite the imaging findings.

Autoimmune diseases such as anti-neutrophil cytoplasmic antibody (ANCA) associated vasculitis were also considered; however, they were deemed less likely in the absence of classic clinical manifestations (ie, sinus involvement, renal dysfunction, neuropathy). However, with nodular disease noted on CT scan and rare case reports of cardiac masses associated with ANCA vasculitis, autoimmune disease was still a consideration.^2^

Malignancy was also in consideration given his relatively asymptomatic presentation. In the setting of malignancy, diffuse pulmonary nodules are more likely secondary to metastatic spread. As such, testicular cancer, soft tissue sarcoma, melanoma, papillary thyroid cancer, renal cell carcinoma, and colon cancer were considered as potential primary sources. A thorough physical examination did not rule out malignancy, but it did make these specific malignant etiologies less likely. CT scan of the abdomen and pelvis was also negative for masses or metastasis. Although primary lung cancers often present with discrete primary lesions, there have been presentations with multifocal micronodular manifestations, such as in metastatic lung adenocarcinoma.^3^ With the right-sided atrial mass and clinical concern for malignancy, there was also suspicion for an invasive vascular tumor such as angiosarcoma.

Throughout the remainder of his hospital course, the patient developed worsening hemoptysis. His oxygen requirement increased steadily, ultimately requiring intubation. Oncology was consulted for potential inpatient therapy, but antiangiogenic therapy could not be initiated because of its contraindication with active hemoptysis. On discussion with a sarcoma specialist, doxorubicin was considered but was thought to provide low likelihood of benefit. After extensive discussion with his family, the decision was made to pursue comfort measures only. The patient was compassionately extubated and died shortly thereafter.

### Radiologic Discussion

#### Chest CT Scan

Although a right-sided atrial mass was present, given that the parenchymal findings of TIB pattern and ground-glass opacities (GGOs) are much more commonly seen with infection, there was consideration for 2 separate processes—incidental right-sided atrial mass with concurrent, unrelated pulmonary infection. TIB pattern occurs when dilated bronchioles are impacted with substances like mucus, pus, or fluid. Although more commonly associated with infection, a TIB pattern is also seen in other airway disorders, such as congenital disorders (ie, cystic fibrosis, Kartagener syndrome), obliterative bronchiolitis, allergic bronchopulmonary aspergillosis, and connective tissue disorders (ie, rheumatoid arthritis, Sjogren syndrome). It has also been reported in malignancies, which occur when tumor cells spread to centrilobular arteries (ie, tumor emboli) or when tumor invasion elicits inflammation of the small pulmonary arteries in carcinomatous endarteritis.^4^

In a study of 18 patients with pulmonary epithelioid hemangioendothelioma (EHE), a TIB pattern was not described. Rather, GGOs were seen in 5 patients (ie, 27.8%). The liver and bone were identified most frequently as extrapulmonary organs affected by EHE.^5^ There have been few other reports of cardiac involvement, namely right-sided atrial masses, also associated with bilateral pulmonary nodules.^6^, ^7^, ^8^ Given the presence of a right-sided atrial mass in the patient and known reports of pulmonary metastasis, it is likely that the presence of a TIB pattern was secondary to vascular embolic spread. GGOs may also represent hemorrhage secondary to intravascular tumor emboli in this patient with a metastatic vascular sarcoma.

#### Cardiac MRI Scan

With late gadolinium enhancement (LGE) imaging, tumors can be differentiated from thrombi. Tumors often show LGE, whereas thrombi do not. Based on their age, thrombi have variable signal on T1- and T2-weighted imaging. Cardiac MRI scan revealed a 2.5- × 4.1-cm mass with high signal on T1-weighted double inversion recovery images and internal heterogeneous LGE. Peripheral areas remained dark on all sequences. This appearance was suggestive of a tumor with surrounding thrombus. Our differential for this right-sided atrial mass included angiosarcoma, cardiac lymphoma, undifferentiated sarcoma, and myxoma.

#### Bronchoscopy

**Figure 4:**
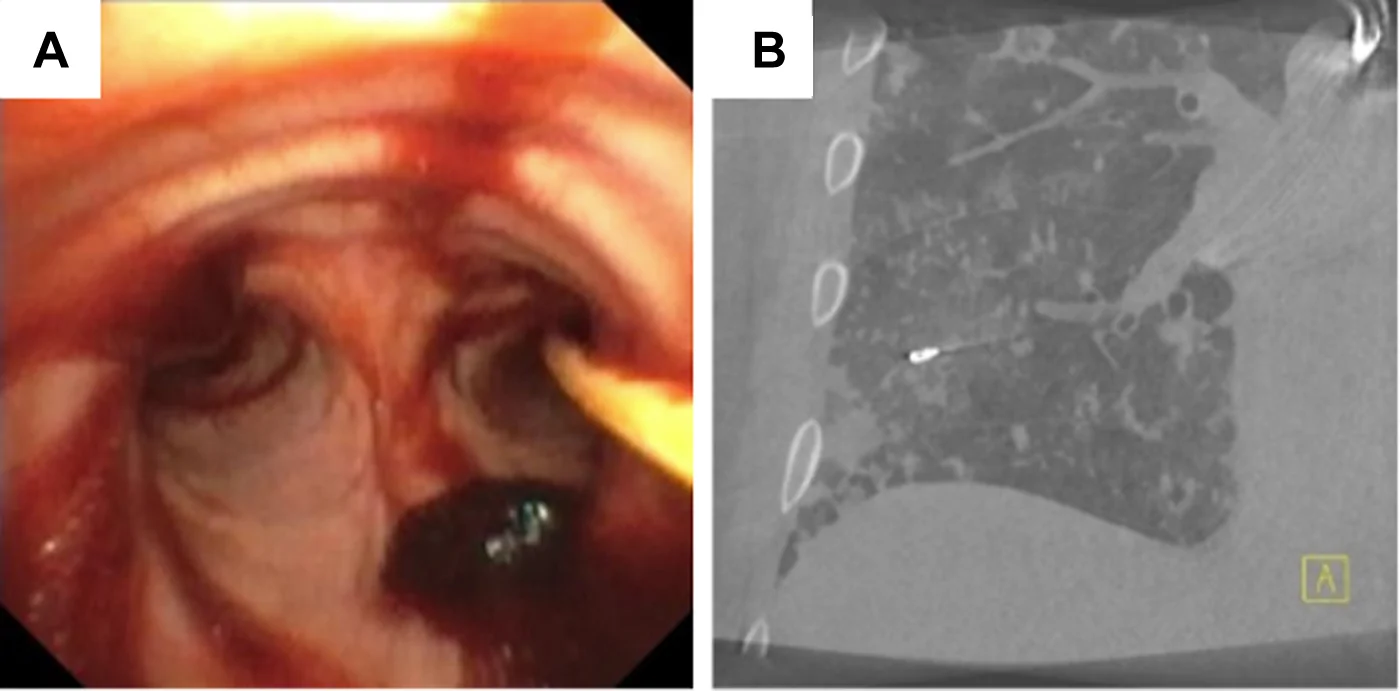
A, Diffuse bleeding was visualized on initial inspection via bronchoscopy. B, Fluoroscopic imaging was used to guide the biopsy.

On initial inspection via flexible bronchoscopy, diffuse bleeding stigmata were noted throughout the bronchial tree (Fig 4A). After therapeutic aspiration of bloody secretions, 3 cryobiopsies were obtained using a 1.7-mm Erbe cryoprobe: 2 from the lateral and posterior segments of the right lower lobe and 1 from the lateral segment of the right middle lobe. Figure 4B demonstrates intraoperative 3-dimensional fluoroscopic imaging used to guide and confirm optimal positioning of the cryoprobe at the right middle lobe biopsy site. Linear endobronchial ultrasound revealed no significant mediastinal lymphadenopathy. Lymph node biopsy was taken from station 4R. Pathology revealed no evidence of malignancy.

### Pathologic Discussion

Sections revealed multiple nodular and infiltrative lesions composed of cords, nests, and short strands of epithelioid cells embedded within a characteristic fibrotic stroma. The tumor cells exhibited round to oval nuclei with vesicular chromatin, occasional nuclear grooves, and small nucleoli. The cytoplasm was moderate in amount, eosinophilic, and variably vacuolated. In some cells, characteristic intracytoplasmic lumina containing erythrocytes (ie, blister cells) were identified.

**Figure 5:**
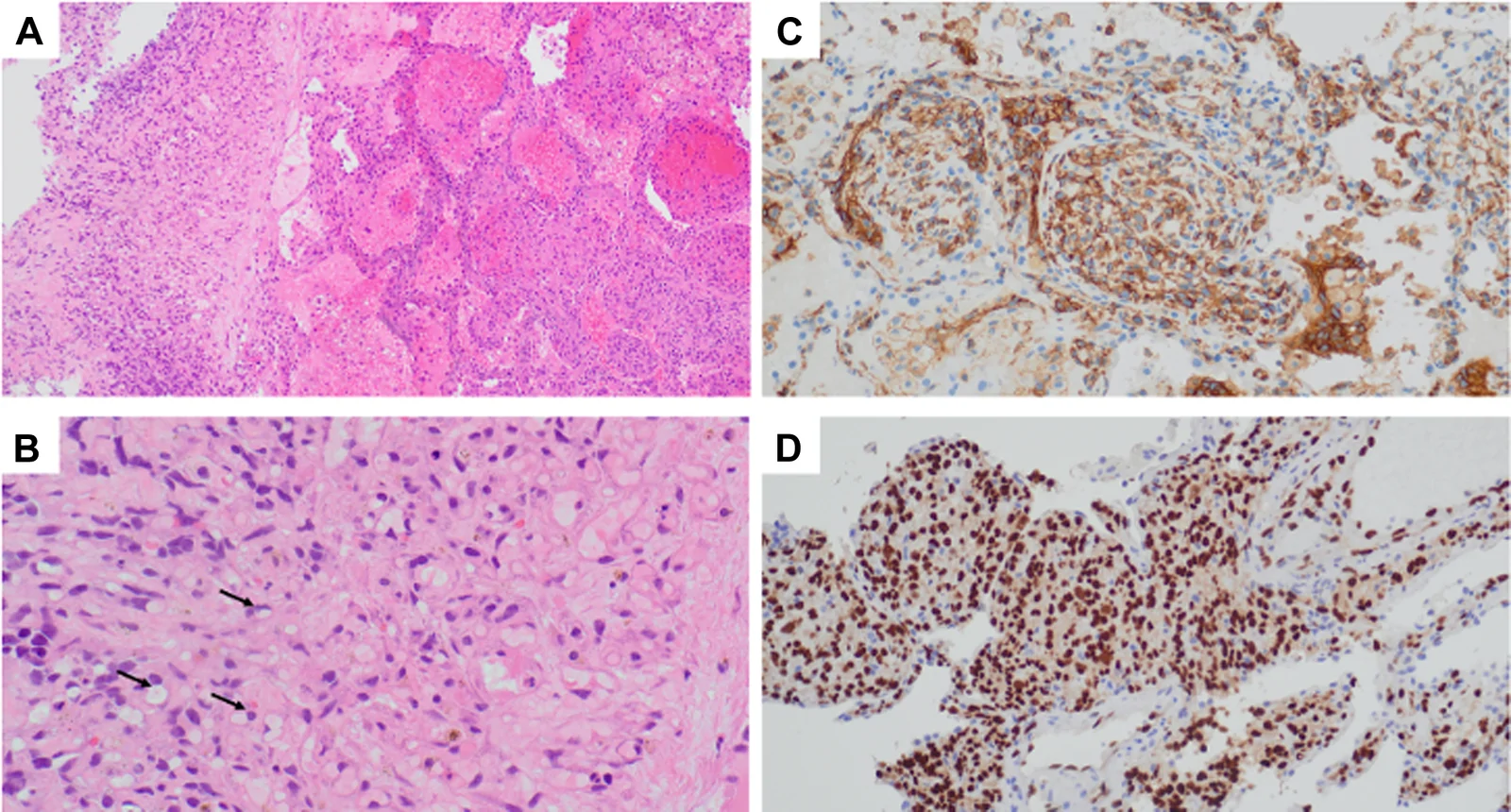
A-D, On hematoxylin and eosin (HE) stain, low-power view at 10x magnification demonstrated an infiltrative growth pattern along alveolar septa and within small vascular channels, with relative preservation of the native pulmonary architecture. In focal areas, the tumor showed intra-alveolar proliferation, with expansion and partial filling of alveolar spaces while maintaining the underlying alveolar framework. Mitotic activity was infrequent, and necrosis was absent or minimal. Occasional angiocentric growth was noted, with neoplastic cells lining vascular lumina A, On HE stain, high-power view at 40x magnification showed tumor cells with round to oval nuclei, vesicular chromatin, and occasional nuclear grooves. Nucleoli were inconspicuous to small. The cytoplasm was moderate, eosinophilic, and variably vacuolated. Characteristic intracytoplasmic lumina containing erythrocytes (“blister cells”) were identified B, Immunohistochemical staining for cluster of differentiation 31 (CD31) demonstrated strong membranous staining of the tumor cells, supporting endothelial differentiation (C). Immunohistochemical staining for erythroblast transformation-specific related gene (ERG) showed nuclear positivity in the tumor cells, further confirming endothelial differentiation (D).

The neoplasm displayed an infiltrative growth pattern along alveolar septa and within small blood vessels, frequently preserving the overall pulmonary architecture. In certain foci, tumor cells expanded and filled alveolar spaces in an intraalveolar growth pattern while maintaining the underlying alveolar framework. Mitotic activity was low, and necrosis was absent or minimal. Focally, the tumor exhibited angiocentric growth, with neoplastic cells lining vascular spaces (Fig 5).

Gene fusion, namely *WWTR1-CAMTA1* fusion, has been associated with EHE.^9^ *CAMTA1* immunostaining was not performed because it was unavailable at both our facility and our reference laboratory. Although *CAMTA1* is recognized as a useful marker for the diagnosis of EHE, the histologic features observed on hematoxylin and eosin staining, together with other vascular markers, were sufficient to establish the diagnosis in this case.

With a prevalence of only 1 in 1 million people, EHE is an exceptionally rare malignancy.^9^ Much of what is known about this malignancy is derived from case reports and retrospective studies.^10^ The median age of presentation is 36 years, more commonly affecting women than men in a 4 to 1 ratio. Approximately one-third of cases present as pulmonary EHE. Other commonly involved sites include the liver and bone.^9^ Although most patients are asymptomatic with diagnoses made after incidental findings on chest imaging, some present with constitutional or organ-specific symptoms (eg, cough, hemoptysis, pleural effusion).^10^ Those presenting with constitutional symptoms, respiratory symptoms, lymph node involvement, pleural involvement, or metastasis tend to have a worse prognosis.^10^ Treatment is variable given the lack of clinical research to inform optimal management. As such, management strategies have ranged from watchful waiting in asymptomatic patients to surgical resection, cytotoxic chemotherapy, immune therapy, and targeted therapy.^9^

## Conclusions

Although chest imaging can be suggestive of an infectious process, in the absence of classical infectious symptoms, alternative diagnoses such as autoimmune conditions or malignancy should be considered. Although the radiologic finding of a TIB pattern is most often associated with an infectious process, it can be a rare manifestation of tumor emboli, especially in the case of vascular tumors. EHE is a rare vascular malignancy, often affecting the lung, liver, bones, and less commonly the heart. Although more commonly associated with an indolent course, EHE has the potential to metastasize and cause rapid decline, as seen in this case. There is no standardized treatment algorithm for EHE, highlighting the need for future studies for this rare but potentially fatal disease.

## Financial/Nonfinancial Disclosures

None declared.
